# Supplementary material for: ComBat-met: adjusting batch effects in DNA methylation data
Source: NAR Genom Bioinform. 2025 May 19;7(2):lqaf062. doi: 10.1093/nargab/lqaf062 (PMC12086544; doi:10.1093/nargab/lqaf062)
Supplement: lqaf062_Supplemental_File [file lqaf062_supplemental_file.pdf]

ComBat-met: Adjusting Batch Effects in DNA Methylation Data

Junmin Wang[1,*]

[1] *Data Sciences and Quantitative Biology, Discovery Sciences, Biopharmaceuticals R&D, AstraZeneca, Waltham, Massachusetts*

[*] *Corresponding author: Junmin Wang (jmwang.bio@gmail.com)*

Supplementary Information

- Supplementary Methods
- Supplementary Figures
- Supplementary References

Supplementary Methods

Parameter Estimation with Shrinkage

Inspired by ComBat and ComBat-seq, we provide a similar non-parametric empirical Bayes method to shrink parameters in ComBat-met (Johnson, Li, and Rabinovic 2007; Zhang, Parmigiani, and Johnson 2020). Let y_{sij} denote the β-value of feature s in sample j from batch i. y_{sij} is assumed to follow a beta distribution, where μ_{sij} and ϕ_{si} denote the mean and precision of the distribution. The beta regression model is defined as:

$$\log\left(\frac{\mu_{sij}}{1-\mu_{sij}}\right) = \alpha_s + X_j\beta_s + \gamma_{si}$$

$$var(y_{sij}) = \frac{\mu_{sij}(1-\mu_{sij})}{1+\phi_{si}},$$

where α_s, β_s, γ_{si}, and ϕ_{si} are defined the same as in the main text for each feature s. In the empirical Bayes framework, the estimated values of γ_{si} and $\ln[\phi_{si}]$ are adjusted to the means of the posterior distributions. Specifically, the empirical Bayes estimates of γ_{si} and $\ln[\phi_{si}]$ are calculated as the weighted average across features:

$$\hat{\gamma}_{si}^{*} = \frac{\sum_{k\neq s}^{K} \omega_{ki}\hat{\gamma}_{ki}}{\sum_{k\neq s}^{K} \omega_{ki}}$$

$$\ln\left[\hat{\phi}_{si}^{*}\right] = \frac{\sum_{k\neq s}^{K} \omega_{ki} \ln\left[\hat{\phi}_{ki}\right]}{\sum_{k\neq s}^{K} \omega_{ki}},$$

where $\hat{\gamma}_{ki}$ and $\ln\left[\hat{\phi}_{ki}\right]$ denote the maximum likelihood estimates of γ_{ki} and $\ln[\phi_{ki}]$, respectively. K is the total number of features, or a subset of features selected to approximate the posterior distribution. The weights ω_{ki} are defined as the beta likelihood functions:

$$\omega_{ki} = \prod_{j=1}^{n_i} d\left(y_{sij}|\hat{\gamma}_{ki}, \hat{\phi}_{ki}\right),$$

where d denotes the density function of the beta distribution, and n_i is the number of samples in batch i. Parameters for batch-free distributions, μ_{sj}^{*} and ϕ_s^{*}, are calculated as:

$$\log\left(\frac{\mu_{sj}^{*}}{1-\mu_{sj}^{*}}\right) = \log\left(\frac{\hat{\mu}_{sij}}{1-\hat{\mu}_{sij}}\right) - \hat{\gamma}_{si}^{*}$$

$$\phi_s^{*} = \frac{\sum_{i=1}^{N_B} n_i\hat{\phi}_{si}^{*}}{\sum_{i=1}^{N_B} n_i}.$$

Supplementary Figures

Beta-binomial regression models

Feature-wise model: methylated count in sample *j* from batch *i*: $y_{ij} \sim BetaBin(T_j, \mu_{ij}, \phi_i)$.

$$\log\left(\frac{\mu_{ij}}{1-\mu_{ij}}\right) = \alpha + X_j\beta + \gamma_i$$

$$Var(y_{ij}) = T_j\mu_{ij}(1-\mu_{ij})\left[1+\frac{T_j-1}{1+\phi_i}\right]$$

α	Baseline level	$X_j\beta$	Sample condition *j*
γ_i	Mean effect of batch i	ϕ_i	Precision of batch i
T_j	Total count in sample *j*		

Estimation of batch effects

Estimate batch effect parameters using beta-binomial regression.

Calculation of "batch-free" distributions

We assume that the post-adjustment data also follow a beta-binomial distribution: $y_j^* \sim BetaBin(T_j, \mu_j^*, \phi^*)$.

$$\log\left(\frac{\mu_j^*}{1-\mu_j^*}\right) = \log\left(\frac{\hat{\mu}_{ij}}{1-\hat{\mu}_{ij}}\right) - \hat{\gamma}_i$$

$$\phi^* = \frac{\sum_{i=1}^{N_B} n_i\hat{\phi}_i}{\sum_{i=1}^{N_B} n_i}$$

Data adjustment

Mapping the data from the estimated distributions to "batch-free" distributions.

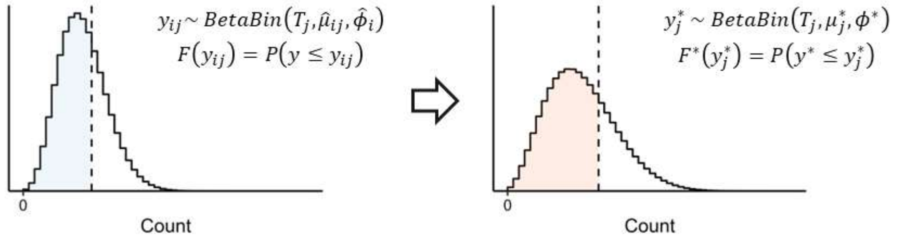

Supplementary Fig. 1. Diagram of the ComBat-biseq workflow.

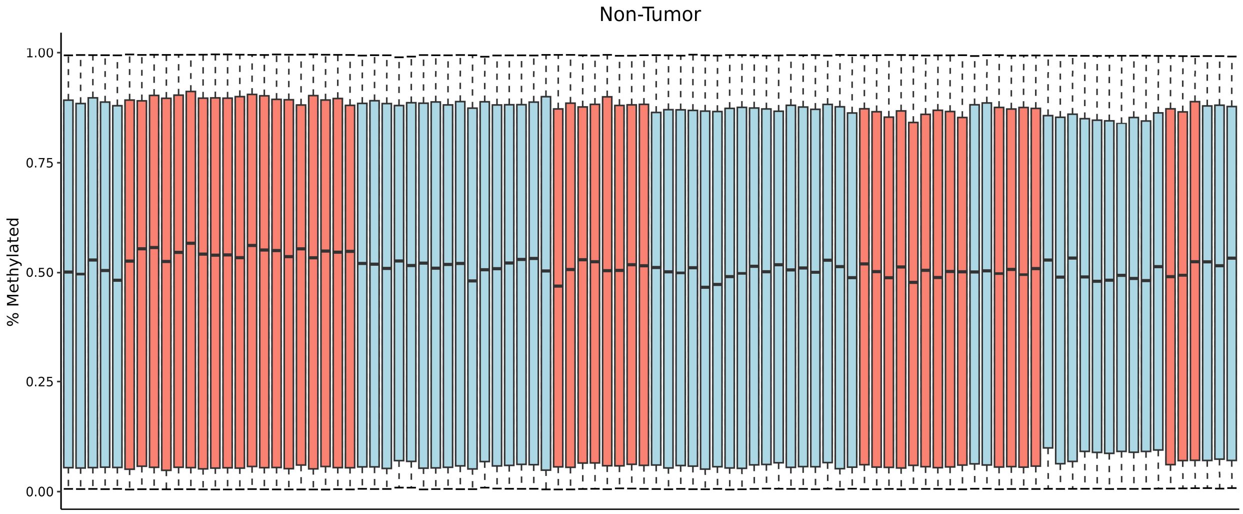

Supplementary Fig. 2. Box plot illustrating the distribution of β-values per sample in the adjacent normal tissues of breast cancer patients. Batches are shown by color.

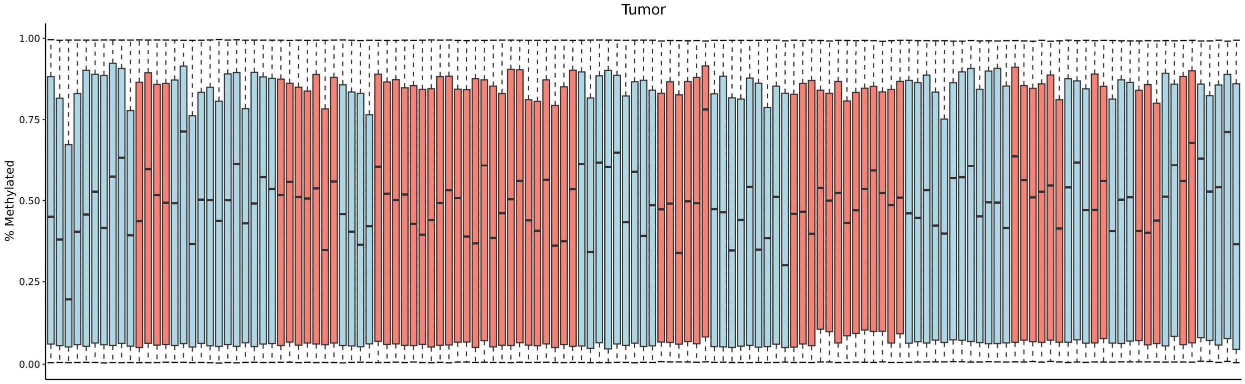

Supplementary Fig. 3. Box plot illustrating the distribution of β-values per sample in the tumor tissues of breast cancer patients. Batches are shown by color.

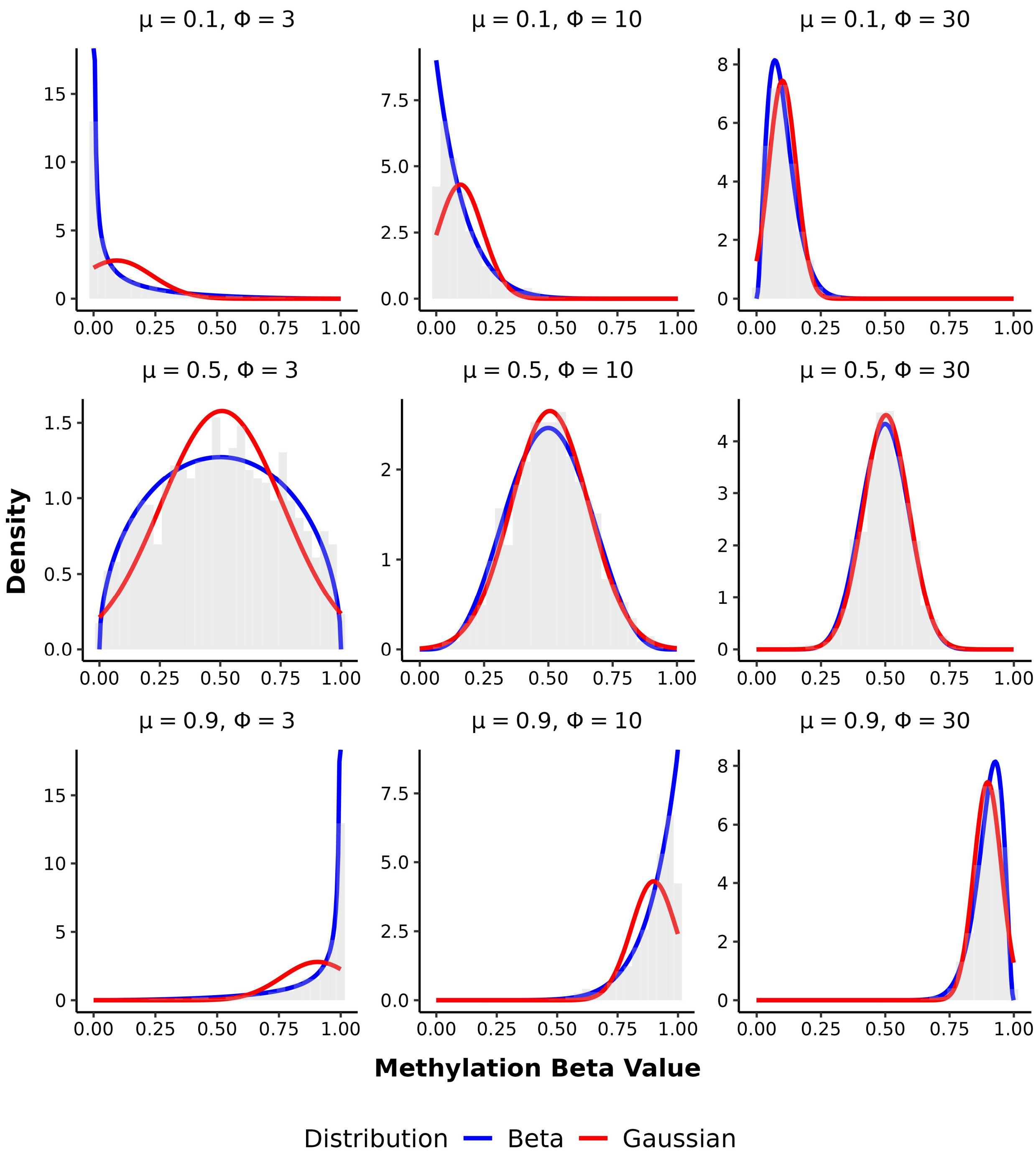

Supplementary Fig. 4. Comparison of beta and Gaussian distribution fits across different parameter settings. Each panel represents data generated from a beta distribution with mean (μ) set to 0.1, 0.5, or 0.9, and precision (ϕ) set to 3, 10, or 30. The shaded gray area shows the histogram of β-values generated, the blue curve represents the theoretical beta distribution, and the red curve depicts the best-fit Gaussian distribution.

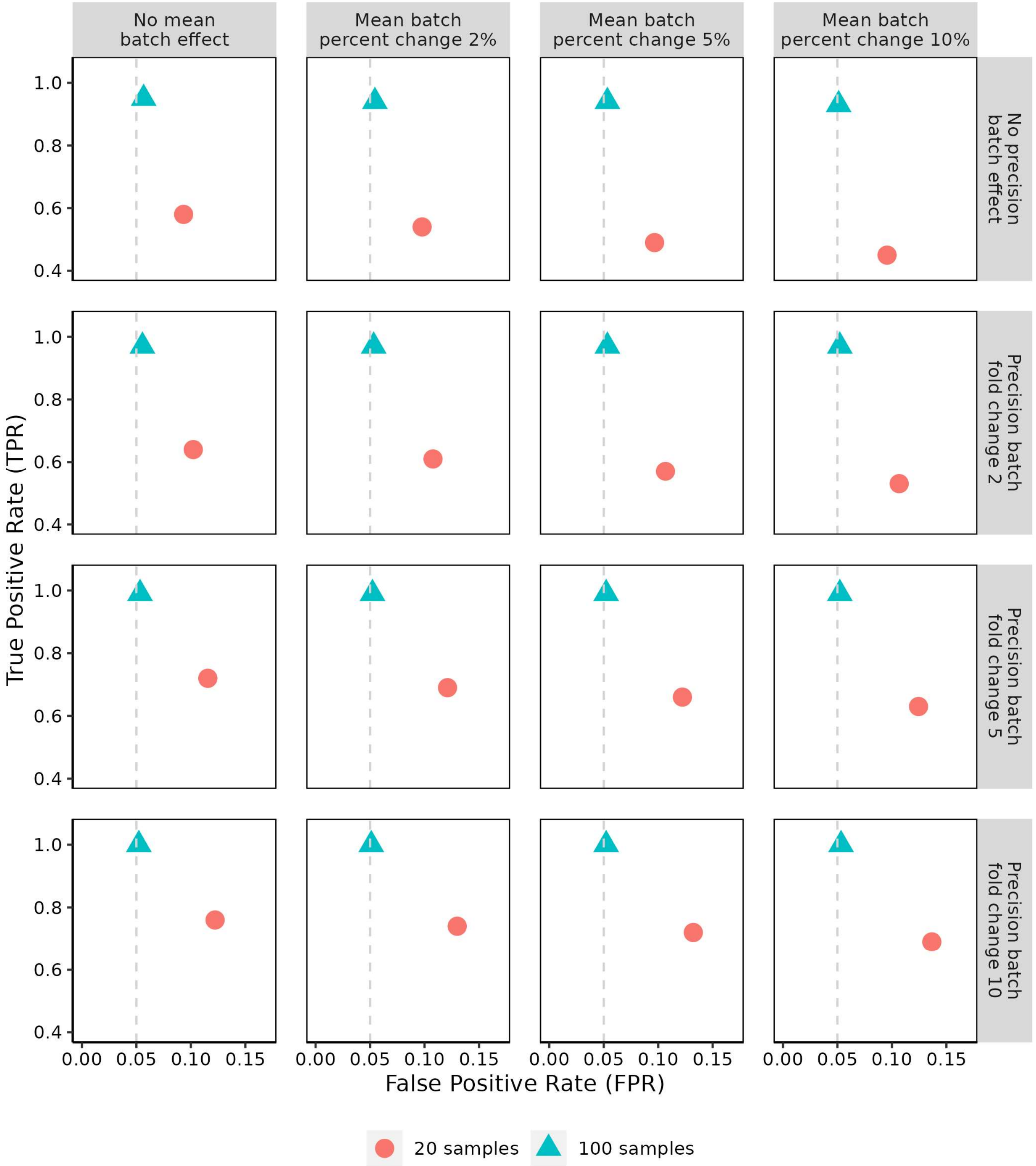

Supplementary Fig. 5. Median true positive rates and false positive rates of ComBat-biseq based on simulation data. Either 20 or 100 samples were simulated. The cross-batch mean difference in methylation percentage was set to 0%, 2%, 5%, or 10%. The precision of the batch effect was set to have a 1-, 2-, 5-, or 10-fold change. The simulation was repeated 1000 times.

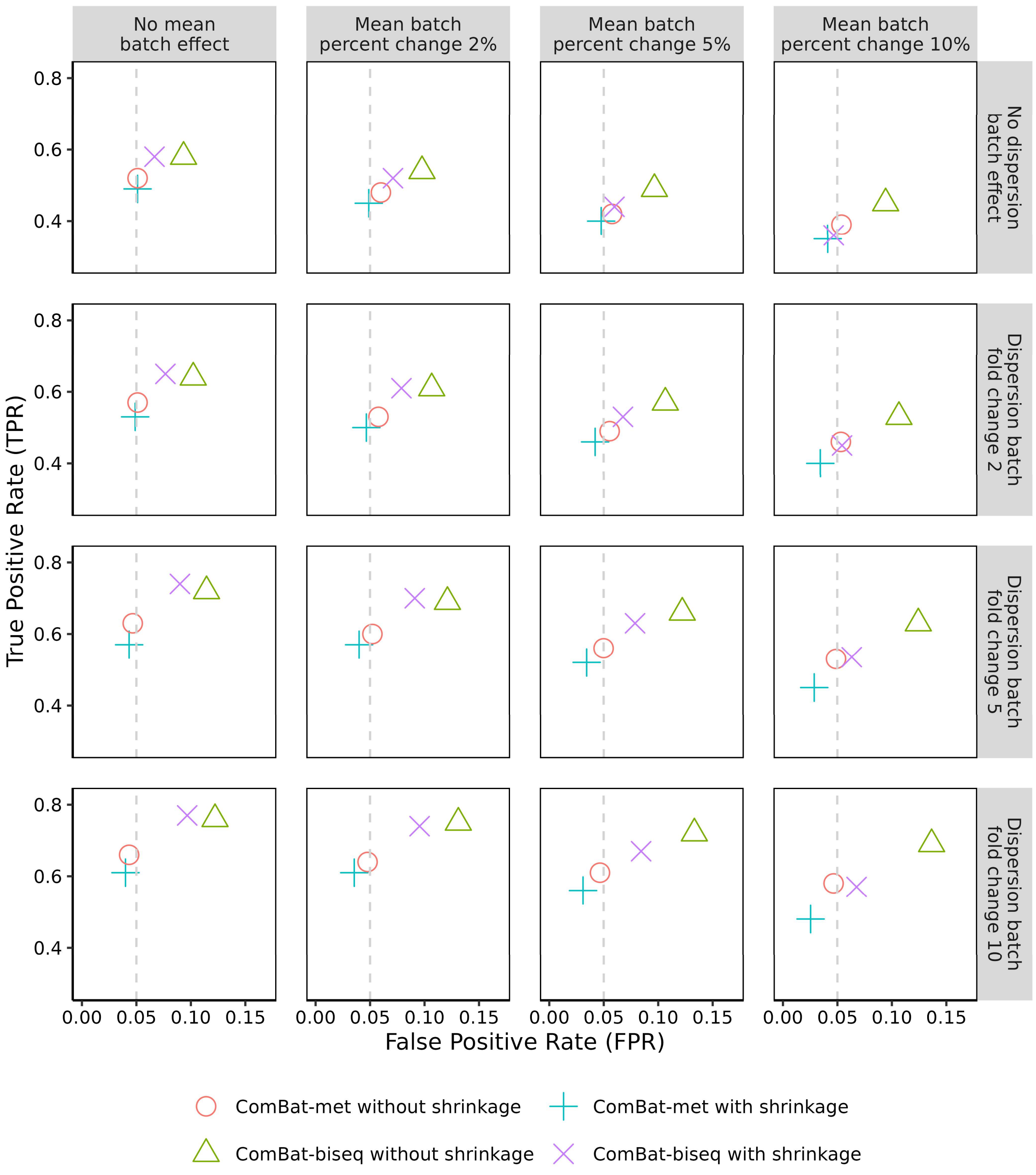

Supplementary Fig. 6. Median true positive rates and false positive rates of ComBat-met and ComBat-biseq with and without parameter shrinkage calculated based on simulation data. The cross-batch mean difference in methylation percentage was set to 0%, 2%, 5%, or 10%. The precision of the batch effect was set to have a 1-, 2-, 5-, or 10-fold change. The simulation was repeated 1000 times. Methods are shown by color.

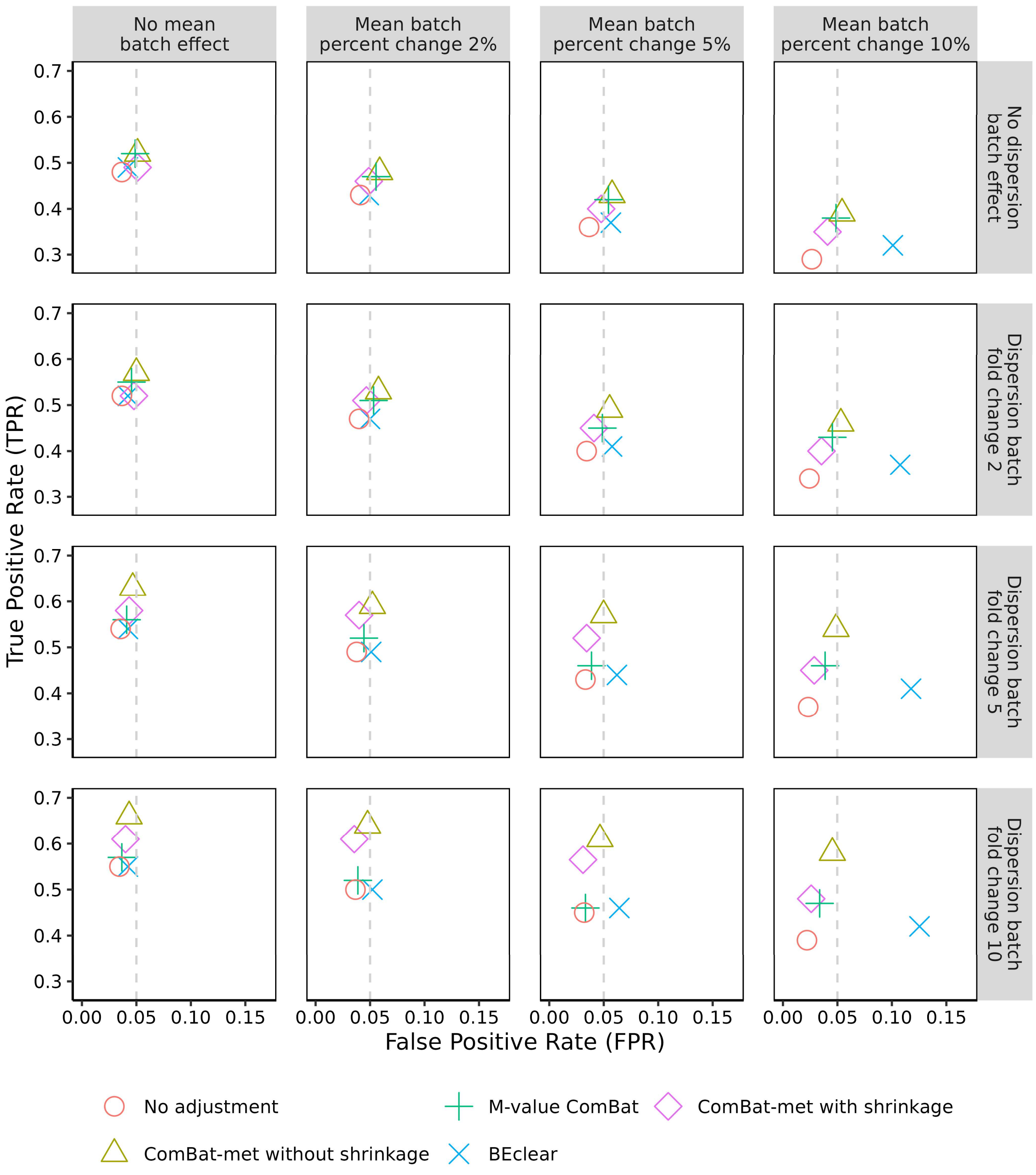

Supplementary Fig. 7. Median true positive rates and false positive rates calculated based on simulation data using no adjustment, M-value ComBat, ComBat-met with shrinkage, ComBat-met without shrinkage, or BEclear followed by minfi. The cross-batch mean difference in methylation percentage was set to 0%, 2%, 5%, or 10%. The precision of the batch effect was set to have a 1-, 2-, 5-, or 10-fold change. The simulation was repeated 1000 times. Methods are shown by color.

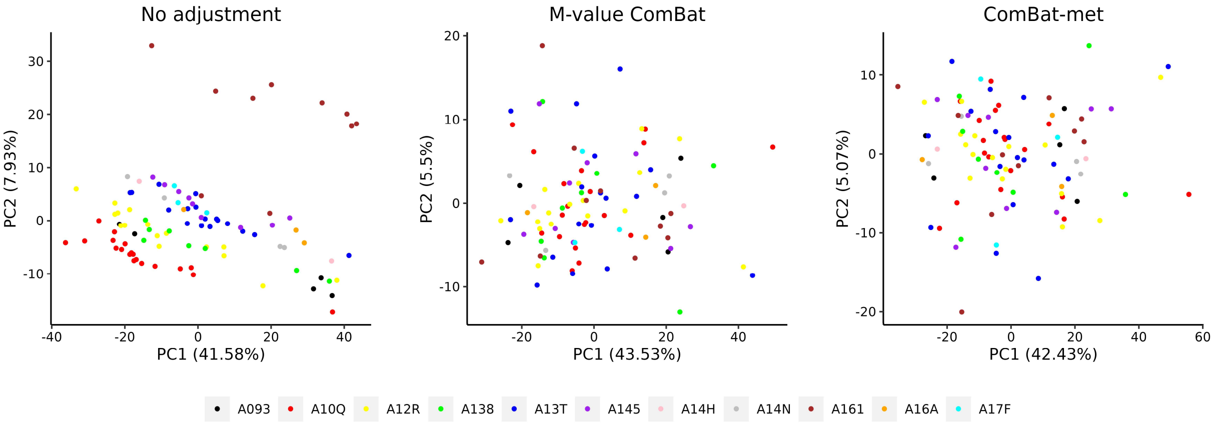

Supplementary Fig. 8. PCA plots illustrating the separation of adjacent normal tissue samples in the unadjusted probe-level data, probe-level data adjusted by M-value ComBat, and probe-level data adjusted by ComBat-met. Batches are shown by color.

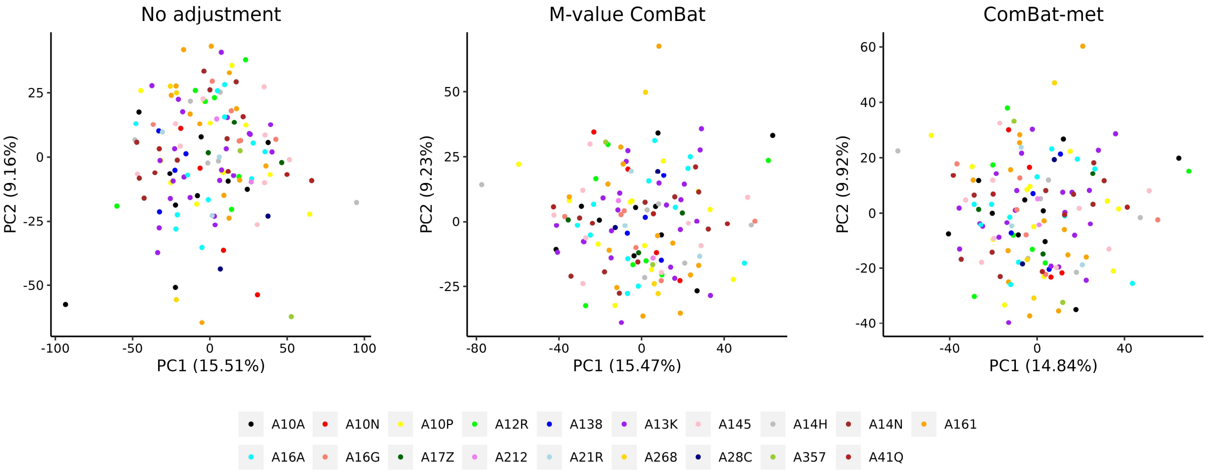

Supplementary Fig. 9. PCA plots illustrating the separation of tumor tissue samples in the unadjusted probe-level data, probe-level data adjusted by M-value ComBat, and probe-level data adjusted by ComBat-met. Batches are shown by color.

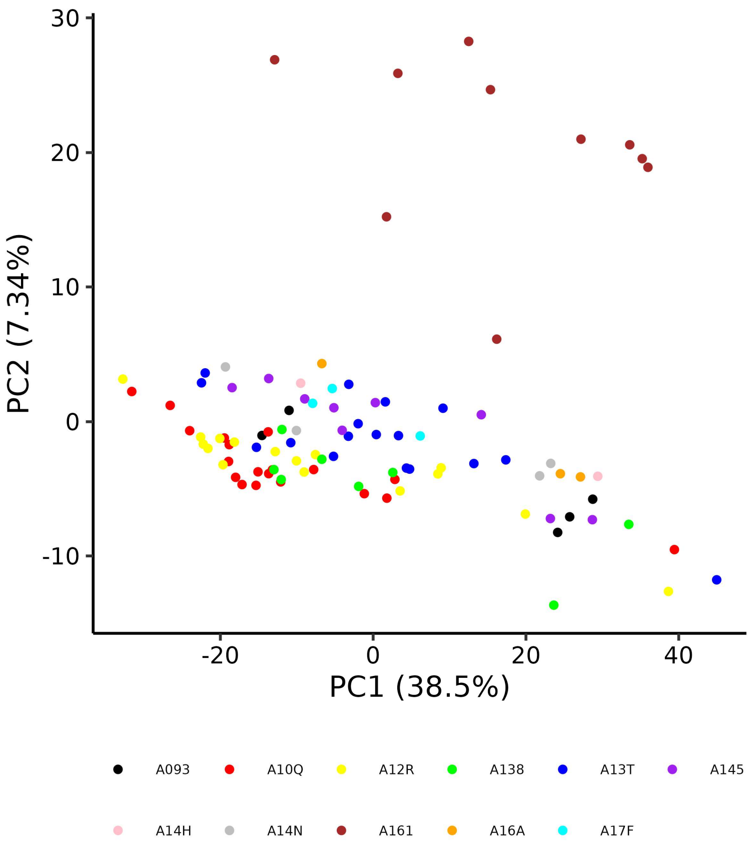

Supplementary Fig. 10. PCA plot illustrating the separation of adjacent normal tissue samples in the data adjusted by ComBat-met with parameter shrinkage. Batches are shown by color.

Supplementary References

Johnson, W. E., C. Li, and A. Rabinovic. 2007. 'Adjusting batch effects in microarray expression data using empirical Bayes methods', *Biostatistics*, 8: 118-27.

Zhang, Y., G. Parmigiani, and W. E. Johnson. 2020. 'ComBat-seq: batch effect adjustment for RNA-seq count data', *NAR Genom Bioinform*, 2: lqaa078.
